# The complete mitochondrial genome of sea cucumber *Stichopus monotuberculatus* (aspidochirotida: Stichopodidae)

**DOI:** 10.1080/23802359.2019.1673244

**Published:** 2019-10-01

**Authors:** Shengping Zhong, Yonghong Liu, Yanfei Zhao, Guoqiang Huang

**Affiliations:** a Institute of marine drugs, Guangxi University of Chinese Medicine, Nanning, China;; b Key Laboratory of Marine Biotechnology, Guangxi Institute of Oceanology, Beihai, China

**Keywords:** Mitochondrial genome, *Stichopus monotuberculatus*, Holothuroidea

## Abstract

The sea cucumber, *Stichopus monotuberculatus*, is an economically important holothuroid in China due to its larger body size and valuable nutrition. However, the taxonomic revision studies of Stichopodidae have been one of the most controversial issues in recent years. Moreover, there remain considerable doubts about a complex of cryptic species within *S. monotuberculatus*. In this study, we report the complete mitochondrial genome of *S. monotuberculatus*. The mitogenome has 16,274 base pairs (60.4% A + T content) and made up of total of 37 genes (13 protein-coding, 22 transfer RNAs and 2 ribosomal RNAs), and a putative control region. This study adds one more available complete mitogenomes of *Stichopus* and will provide useful genetic information for future evolutionary and taxonomic classification of Stichopodidae.

Holothuroids, or sea cucumbers, is a diverse and abundant group of ecologically and economically important benthic organism, which includes more than 1400 species around the world’s oceans from the deep sea trench to intertidal zones (Shen et al. 2009; Mu et al. 2018). The sea cucumber *Stichopus monotuberculatus*, widely distributed throughout the Indo-west Pacific region, is a commercially important tropical species in China which was known as a delicious seafood and a traditional medicine for its valuable nutrition. However, despite their great diversity and ecological importance, there remain considerable doubts about phylogenetic relationships and evolution in Holothuroidea (Perseke et al. 2010). The incongruences between morphology based taxonomic systems and molecular systematic has been debated recently(Fan et al. 2012). The complete mitochondrial genome is an excellent molecular marker for studying phylogenetic relationships and species identification (Shen et al. 2009; Mu et al. 2018). Here, we report the complete mitochondrial genome sequence of *S. monotuberculatus*, which will provide a better understanding of higher-level relationships within Holothuroidea.

A tissue samples of *S. monotuberculatus* from 3 individuals were collected from GuangXi province, China (Beihai, 21.051398 N, 108.944452 E), and the whole body specimen (#GH0047) were deposited at Marine biological Herbarium, Guangxi Institute of Oceanology, Beihai, China. The total genomic DNA was extracted from the muscle of the specimens using an SQ Tissue DNA Kit (OMEGA, Guangzhou, China) following the manufacturer’s protocol. DNA libraries (350 bp insert) were constructed with the TruSeq NanoTM kit (Illumina, San Diego, CA) and were sequenced (2 × 150bp paired-end) using HiSeq platform at Novogene Company, China. Mitogenome assembly was performed by MITObim (Hahn et al. 2013). complete mitogenome of *Stichopus horrens* (GenBank accession number: NC_014454) was chosen as the initial reference sequence for MITObim assembly. Gene annotation was performed by MITOS (Bernt et al. 2013).

The complete mitogenome of *S. monotuberculatus* was 16,274 bp in length (GenBank accession number: MN276189), and containing the typical set of 13 protein-coding, 22 tRNA and 2 rRNA genes, and a putative control region. The overall base composition of the mitogenome was estimated to be A 31.0%, T 29.4%, C 23.6% and G 16.0%, with a high A + T content of 60.4%, which is similar, but slightly higher than *S. horrens* (60.1%). The mitogenomic phylogenetic analyses showed that *S. monotuberculatus* was first clustered with *S. horrens* in the Stichopodidae clade (Figure 1), which is consistent with the phylogenetic analyses of Stichopodidae using partial COI genes from mitochondrial DNA (Fan et al. 2012). Our mitogenome data supported the sister relationship of *S. monotuberculatus* and *S. horrens*. The complete mitochondrial genome sequence of *S. monotuberculatus* add one more mitogenome of Stichopodidae, which will contribute to further phylogenetic and taxonomic relationships studies of Stichopodidae, and related family.

**Figure 1.**
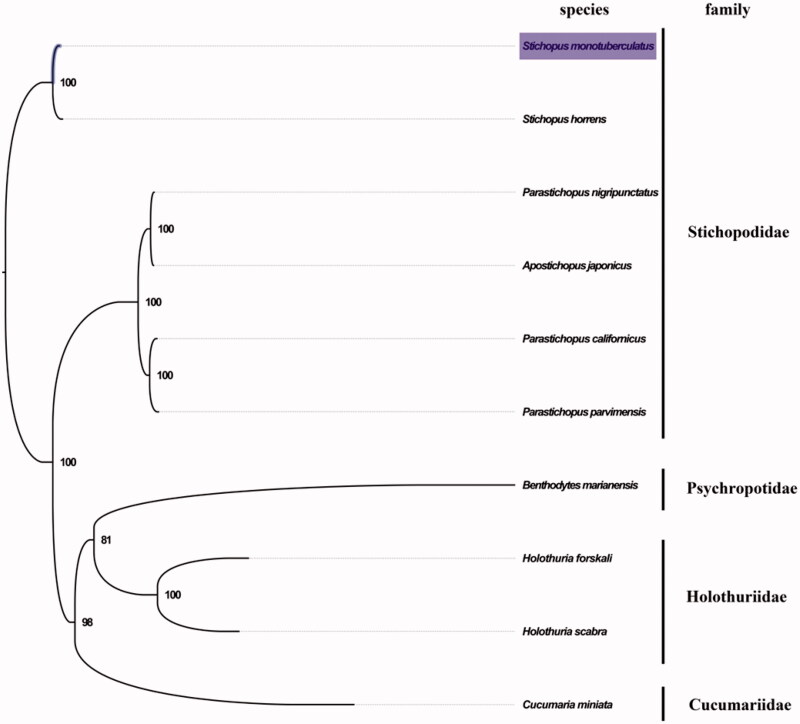
Phylogenetic tree of 10 species in Holothuroidea. The complete mitogenomes is downloaded from GenBank and the phylogenic tree is constructed by maximum-likelihood method with 100 bootstrap replicates. The bootstrap values were labeled at each branch nodes. The gene's accession number for tree construction is listed as follows: *Stichopus horrens* (NC_014454), *Parastichopus nigripunctatus* (NC_013432), *Apostichopus japonicus* (NC_012616), *Parastichopus californicus* (NC_026727), *Parastichopus parvimensis* (NC_029699), *Benthodytes marianensis* (NC_040968), *Holothuria forskali* (NC_013884), *Holothuria scabra* (NC_027086), and *Cucumaria miniata* (NC_005929).
